# Altered topology of structural brain networks in patients with Gilles de la Tourette syndrome

**DOI:** 10.1038/s41598-017-10920-y

**Published:** 2017-09-06

**Authors:** E. Schlemm, B. Cheng, F. Fischer, C. Hilgetag, C. Gerloff, G. Thomalla

**Affiliations:** 1 Department of Neurology, University Medical Center Hamburg-Eppendorf, Martinistraße 52, 20246 Hamburg, Germany; 2 Department of Computational Neuroscience, University Medical Center Hamburg, Martinistraße 52, 20246 Hamburg, Germany

## Abstract

Gilles de la Tourette syndrome is a neurodevelopmental disorder characterized by tics. Abnormal neuronal circuits in a wide-spread structural and functional network involved in planning, execution and control of motor functions are thought to represent the underlying pathology. We therefore studied changes of structural brain networks in 13 adult GTS patients reconstructed by diffusion tensor imaging and probabilistic tractography. Structural connectivity and network topology were characterized by graph theoretical measures and compared to 13 age-matched controls. In GTS patients, significantly reduced connectivity was detected in right hemispheric networks. These were furthermore characterized by significantly reduced local graph parameters (local clustering, efficiency and strength) indicating decreased structural segregation of local subnetworks. Contrasting these results, whole brain and right hemispheric networks of GTS patients showed significantly increased normalized global efficiency indicating an overall increase of structural integration among distributed areas. Higher global efficiency was associated with tic severity (R = 0.63, p = 0.022) suggesting the clinical relevance of altered network topology. Our findings reflect an imbalance between structural integration and segregation in right hemispheric structural connectome of patients with GTS. These changes might be related to an underlying pathology of impaired neuronal development, but could also indicate potential adaptive plasticity.

## Introduction

Gilles de la Tourette (GTS) syndrome is a complex developmental neuropsychiatric disorder with childhood onset^1^. Tics are the clinical hallmark and defining feature of GTS. They are defined as hyperkinetic movements that resemble voluntary actions but are out of context and often exaggerated. Tics are highly variable in their phenomenology and often accompanied by premonitory sensations or urges as an emotional component. Tics are often perceived as disruptive and socially inacceptable, and many GTS patients are able to control and suppress them to a certain degree. The aetiology of GTS and specifically tics as its core symptom still remains unknown. However, a common pathophysiological framework has been established based on clinical observations, neuroanatomical studies and results from structural and functional neuroimaging. At its core, disturbances of cortico-striatal-thalamo-cortical (CSTC) loops and abnormally altered dopaminergic neurotransmission have been found to be responsible for tic generation^2–6^. Beyond the classical model of CSTC circuits, an abundance of grey and white matter alterations have been found in GTS patients in widely distributed cortical and subcortical areas^7–9^ relating to tic generation. In addition, extended abnormalities and disorganized activation beyond sensorimotor regions have been detected by functional MRI in language, paralimbic, insular or executive circuits^10–12^. Taken together, GTS represents a model disorder characterized by abnormal structural and functional networks involved in planning, execution and control of motor actions.

Due to their inherent complexity, brain networks are difficult to compare between individuals or groups. The multitude of potentially altered individual connections imposes significant methodological and statistical restrains. One approach, which has become popular over the last decade, is to model structural brain connectomes that capture the global network architecture and topology^13^. Using graph theory, a set of parameters or network measures^14^ are calculated based on data from diffusion tensor imaging and white matter tractography characterizing individual pathways among a set of cortical and subcortical regions of interest. Graph theoretical measures therefore quantify specific organizational properties of its structure. Their diverging values in different populations are indicative of a difference in the topology of the underlying networks. Structural connectome analysis by graph-theoretical approaches has successfully been applied to study neurological, developmental and psychiatric disorders. Abnormal structural and functional connectivity has been reported in patients with Alzheimer’s disease and mild cognitive impairment, schizophrenia, multiple sclerosis, high-functioning autism and ADHD^15–17^. In all cases, a disturbed balance of network integration and segregation that is characteristic for optimal brain network function has been observed^18^. Network analysis of functional MRI data in patients with GTS and comorbid obsessive compulsive disorder (OCD) has previously revealed an altered global network topology showing patterns of disorganization and “functional immaturity” that point to an abnormal trajectory of brain maturation^19^. Alterations in network organization detected by functional MRI in healthy GTS patients that resembled the topology of brain networks in younger healthy individuals point in a similar direction^20^. Taken together, these findings lend support to the concept of GTS as a prototype neurodevelopmental disorder of brain immaturity leading to disturbed motor and behavioural control.

Up to date, there are only few studies analysing white matter integrity or connectivity in patients with GTS. Applying diffusion tensor imaging (DTI), pathological changes have been detected in individual, pre-defined CSTC circuits^9, 21–24^. None of the previous studies assessed global changes of structural network integrity and topology by graph theoretical analysis. In this study, we therefore reconstructed the structural connectome of 13 adult GTS patients and 13 age- and gender-matched, healthy participants for graph theory based structural brain network analysis. Only patients without comorbidities and currently not treated with neuroleptic medication were included to avoid confounding factors. We hypothesised that distinctive changes in the topology of global white-matter networks could be detected in patients with GTS, reflecting primary, neurodevelopmental pathology as well as potential adaptive plasticity of the structural brain network architecture.

## Materials and Methods

### Subjects

We initially aimed to analyse 15 patients previously included in a study of structural connectivity using probabilistic tracking in a predefined network of motor control^24^. Of those, two patients had to be excluded due to insufficient quality of the structural MRI data precluding complete segmentation of cortical parcellations. Tic severity was rated using the Yale Global Tic Severity Scale (YGTSS) (Leckmann *et al*.^25^). In total, 13 patients (13 men, mean age: 34.5; SD ± 8.9 years, mean YGTSS: 40.2 ± 16.6) were included in the final dataset. The control group comprised age- and gender-matched healthy subjects (13 men, mean age: 34.6; SD ± 9.1 years Clinical assessment was performed by a neurologist or psychiatrist experienced in diagnosing and treating GTS patients. Lifetime clinical information was systematically collected using standardized clinical assessment and a semi-structured interview adapted from Robertson and Eapen^26^ in which patients are systematically screened for premonitory urges and other sensory phenomena, disturbances of social behaviour, impulse control disorder, as well as symptoms of depression or anxiety disorder. GTS and ADHD were diagnosed according to DSM-IV-TR criteria. Patients fulfilling criteria of OCD, ADHD or other co-morbidities were excluded from the study. The study was approved by the local Ethics Committee (Ärztekammer Hamburg, No. 2514) and written informed consent was obtained from all participants prior to the experiment according to the Declaration of Helsinki. All methods were carried out in accordance with relevant guidelines and regulations.

### Image acquisition

We performed MRI at 3 Tesla field strength using a Magnetom Trio TIM (Siemens, Erlangen, Germany) equipped with a gradient system providing a maximum strength of 40mT/m using an 8-channel head coil. DTI data was measured with an echo planar imaging (EPI) whole brain sequence. The sequence was configured as follows: TE/TR = 105/18.500 ms, bandwidth = 1954 Hz/Px, 128 × 128 matrix, FOV 256 × 192 mm^2^, 60 axial slices, 2 mm slice thickness without inter-slice gap, resulting in an isotropic voxel size of 2 × 2 × 2 mm^3^, gradient pulses along 24 different directions with a b-value of 1000 s/mm^2^. Non-diffusion weighted image (b = 0 s/mm^2^) were acquired after every eighth image to guide registration of individual diffusion images. Measuring of DTI data was repeated to increase signal-to-noise ratio resulting in a total scanning time of 16 min 50 s. Structural imaging was performed using a T1-weighted imaging of the whole brain. The Fast Low Angle Shot (FLASH) 3D sequence was configured as follows: TE/TR = 4.92/15 ms, flip angle 25, 192 slices, 1 mm slice thickness, 20% gap, 256 × 256 matrix, FOV 256 × 256 mm^2^. Heads of participants were stabilized using foam pads to minimize movement artifacts. For each subject, measurements of head displacement were derived from realignment parameters.

### Data preprocessing and cortical parcellation

Structural connectome reconstruction was based on a standard freesurfer parcellation scheme and probabilistic tractography as described previously^27^. In brief, diffusion-weighted images were analysed using the FSL software package 5.1 (http://www.fmrib.ox.ac.uk/fsl). All datasets were corrected for eddy currents and head motion. Structural T1-weighted anatomical images were processed using the freesurfer software package 5.3.0 with standard procedures and parameters resulting in a cortical parcellation of 33 cortical and three subcortical regions (thalamus, lentiform and caudate nucleus) per hemisphere^28, 29^ as listed in Table 1. Details of automated anatomical segmentation and labelling according to the Desikan-Killiany Atlas as implemented in freesurfer have been published previously^30^. In total (both hemispheres), 72 regions of interest were created. Volumes of cortical grey matter were calculated using freesurfer and compared using a repeated-measures ANOVA with within-subject factor *region* = {left hemisphere, right hemisphere} and between-subject factor *group* = {controls, patients}. Accuracy of cortical parcellation was checked visually. Each cortical parcellation was transformed to diffusion space using the non-linear transformation coefficient file and accuracy of registration was checked individually.

**Table 1:** Cortical parcellation scheme applied for connectome generation according to the standardized freesurfer algorithm^30^.

Banks superior temporal sulcus
Caudal anterior-cingulate cortex
Caudal middle frontal gyrus
Caudate nucleus
Cuneus cortex
Frontal pole
Fusiform gyrus
Inferior parietal cortex
Inferior temporal gyrus
Insula
Isthmus–cingulate cortex
Lateral occipital cortex
Lateral orbital frontal cortex
Lentiform nucleus
Lingual gyrus
Medial orbital frontal cortex
Middle temporal gyrus
Parahippocampal gyrus
Paracentral lobule
Pars opercularis
Pars orbitalis
Pars triangularis
Pericalcarine cortex
Postcentral gyrus
Posterior-cingulate cortex
Precentral gyrus
Precuneus cortex
Rostral anterior cingulate cortex
Rostral middle frontal gyrus
Superior frontal gyrus
Superior parietal cortex
Superior temporal gyrus
Supramarginal gyrus
Temporal pole
Thalamus
Transverse temporal cortex

Processing of diffusion data included application of a probabilistic diffusion model modified to allow estimation of multiple (n = 2) fiber directions using the program bedpostx. From each seed ROI voxel, 5000 streamlines were initiated through the probability distribution on principle fiber direction. Structural connectivity between two regions was measured by masking each seed ROI results by each of the remaining ROIs.

### Construction of network matrices

Probabilistic tractography provided, for each subject, and each pair $(s,t)\,$of 72 prespecified ROIs, the number of reconstructed fibre tracts starting at *s* and running through *t*. These raw streamline counts were normalised by the product of the volume of the seed and target ROIs, assembled into numerical arrays of size 72-by-72, and symmetrised. Each resulting connectivity matrix was interpreted as the weighted adjacency matrix of a graph, summarising the undirected structural topology of the underlying brain network. In order to explore the localization of anatomical network alterations submatrices corresponding to left and right hemispheres were extracted and analysed separately.

As there is no established threshold for creating connectivity matrices from probabilistic fiber tracking, we aimed at analysing connectivity patterns across a broad range of thresholds. For a given thresholding parameter $\kappa$, binary masks were defined to contain the strongest$\,(100\times \kappa ) \% \,$connections in a summary matrix whose elements represented median connectivity strengths across all subjects, and applied to both controls and patients; the same mask was applied to both groups in order not to artificially inflate any group difference. Thus, by construction, $\kappa \,$ corresponds directly to the edge density in the thresholded networks. The original edge weights were preserved in the thresholded matrices, and no binarization was performed. The thresholding level $\kappa \,$was varied between 0.05 and 0.95 with increments of 0.05 to prevent, on the one hand, excessive fragmentation of the brain networks, and to ensure, on the other hand, that non-anatomical connections that were introduced spuriously by the probabilistic tracking algorithm, were eliminated. The total number of edges thus varied between 128 and 2428 in the whole-brain, and 32 and 599 in the single-hemisphere networks. By examining a range of cut-offs, our results are less sensitive to the effects of false positive connections which might confound the analysis at a single network density.

### Graph theory

We focussed our analysis on the three commonly-used measures global efficiency, clustering coefficient, and modularity, all of which are implemented in the Brain Connectivity Toolbox (BCT) for Matlab^31^. Global efficiency was chosen over the similar characteristic path length because of its more robust calculation.

Global efficiency is regarded as a measure of integration and distant information transfer in a network. In functional brain networks, global efficiency provides a measure of the overall capacity for information transfer and integrated processing among distributed components of the system^14^. More formally, it is defined as the average inverse (shortest) distance between all pairs of nodes in the network. In symbols, the global efficiency can be written as

1 $$\varepsilon =\frac{1}{n(n-1)}\sum _{s\ne t}d{(s,t)}^{-1};\,d(s,t)=\mathop{\min }\limits_{{\rm{p}}:{\rm{s}}\leftrightarrow {\rm{t}}}\sum _{e\in p}{l}_{e}.$$

Here, $n$ is the number of nodes in the network, $p$ is a path connecting the nodes $s$and $t$, and ${l}_{e}={w}_{e}^{-1}$ denotes the length of the edge $e$, given by the inverse connectivity strength.

The global clustering coefficient measures the topological segregation of a network by quantifying the extent to which neighbours of an average node are connected to each other.

For weighted networks it can be expressed mathematically as

2 $$C=\frac{1}{n}\sum _{v\in V}{C}_{v};\,{C}_{v}=\frac{1}{{k}_{v}({k}_{v}-1)}\sum _{x,y\in V}{({w}_{vx}{w}_{vy}{w}_{xy})}^{\frac{1}{3}},$$

where$\,{k}_{v}=\sum _{x\in V}{w}_{vx}\,$denotes the strength of the vertex $v$ and$\,{C}_{v}\,\,$is its local clustering coefficient.

For a given partition of the nodes of a network into disjoint subsets, its modularity is a measure of the extent to which nodes are connected to other nodes in the same subset rather than to nodes in different subsets. The modularity of the network is defined as the optimal value over all possible partitions and can be approximated efficiently by numerical algorithms that avoid the need to enumerate all possible partitions^32^.

The global efficiency and clustering coefficient are affected by both the topology of the network and the strength of connections. It follows from the definitions that, ceteris paribus, larger values of$\,{w}_{e}$, i.e. stronger connections, lead to greater efficiency and a higher clustering coefficient. The corresponding confounding effect of overall connectivity strength in group comparisons can be eliminated by dividing these network measures by their expected value in a random reference network of the same size with the same degree and weight distribution^33^. Modularity is not affected by a global shift in connectivity strengths and was therefore not normalised.

Local features of a network’s topology can be described using nodal graph parameters. The strength of a node is the sum of the weights of its incident edges; the local efficiency of a node is defined as the global efficiency of the subgraph induced by the node and its neighbours. No normalisation was performed for local network measures.

### Statistical analysis

#### Head motion

Head motion is known to have the potential to affect morphometric and diffusion-derived estimates of brain structures and to induce spurious group-differences in graph-theoretical connectivity measures^34, 35^. To address these concerns, the amount of head motion of each subject during image acquisition was quantified by extracting estimates of mean absolute and relative translation along and rotation around three orthogonal spatial directions from registration matrices. These twelve standard parameters were analysed for between-group differences and correlation with averaged connectivity values.

#### Overall connectivity

In a first analysis, motivated by our previous work^24^, the hypothesis of a structural connectivity deficit in Tourette patients was re-investigated by the novel statistical approach of graph theoretical structural network analysis. This analysis extends our initial investigation by definition of a global structural connectome exceeding the previously defined, highly localized network of sensorimotor brain areas. In addition to being informative in its own right, knowledge of the underlying connectivity strength is also a crucial prerequisite for interpreting more topology-oriented weighted network measures. Quartiles of within-subject connectivity strength distributions were computed and compared between groups using a non-parametric multiple permutation test^36^. Equality of group-averaged within-subject connectivity strength distributions was assessed using two-sample Kolmogorov-Smirnov tests. In addition, using the Matlab Toolbox Measures of Effect Size^37^, a two-way repeated measures ANOVA with within-subject factor *region* = {left hemisphere, interhemispheric, right hemisphere} and between-subject factor *group* = {controls, patients} was performed for median connectivity strengths. Magnitudes of effects were assessed quantitatively by partial ${\eta }^{2}$ and Hedges’$g$, which is a measure of the standardized mean-difference between two groups and has reduced bias in small samples compared to the more frequently used Cohen’s $d$.

#### Global graph parameters

For a discrete set of thresholding levels$\,\kappa$, ranging from 0.05 to 0.95, three whole-brain global graph parameters were computed using BCT. In order to control for the effect of different overall connectivity strength and to thus isolate information about the networks’ topology, the global efficiency and clustering coefficient were normalised with respect to their average value in 150 independent realisations of size-, degree- and strength-preserving randomised null models^38^. Modularity was not normalised. Statistical significance of group differences for these three measures was assessed using the multiple threshold cluster permutation (MTCP) approach^39^. This method overcomes the bias inherent in selecting a thresholding level by implementing a permutation test for the supercritical area-under-the-curve, which incorporates both the strength of an effect and its persistence across thresholds. Therefore, it does not suffer from type-I error rate inflation associated with multiple testing across thresholds.

The same global graph parameters were computed for subnetworks corresponding to left and right hemispheres separately and compared between groups using the MTCP approach.

Clinical significance of any observed group differences was investigated by computing linear correlation coefficients between network measures and YGTSS as a marker of disease severity, with and without age as a covariate. Localisation of structural differences to brain regions

To investigate localization of structural and topological network changes, the local network measures strength, efficiency, and clustering coefficient were computed for each brain region at different thresholds. Statistical significance and effect size of group differences across thresholds were quantified with MTCP (see Drakesmith 2015, Fig. 11). More precisely, $n={10}^{3}$ random group reassignments were generated and independent-samples t-tests were performed for each threshold. The 95-percentile of the maximal group effect across thresholds was defined as the critical $t$-value ${t}_{crit}$. A significant group difference at level 5% was declared if the maximal empirical t-statistic across thresholds exceeded${t}_{crit}$. In this case, their associated supercritical area-under-the-curves denoted by A, were computed as an additional measure of effect size and compared to the average scAUC of the random permutations, denoted by${A}_{crit}$.

The datasets generated during and/or analysed during the current study are available from the corresponding author on reasonable request.

## Results

### Head motion

In a linear model, motion parameters did not significantly predict within-subject median connectivity values (${F}_{13,13}=1.715$, $p=0.172$), and correction for motion was therefore not performed for further analysis.

### Overall connectivity

**Figure 1:**
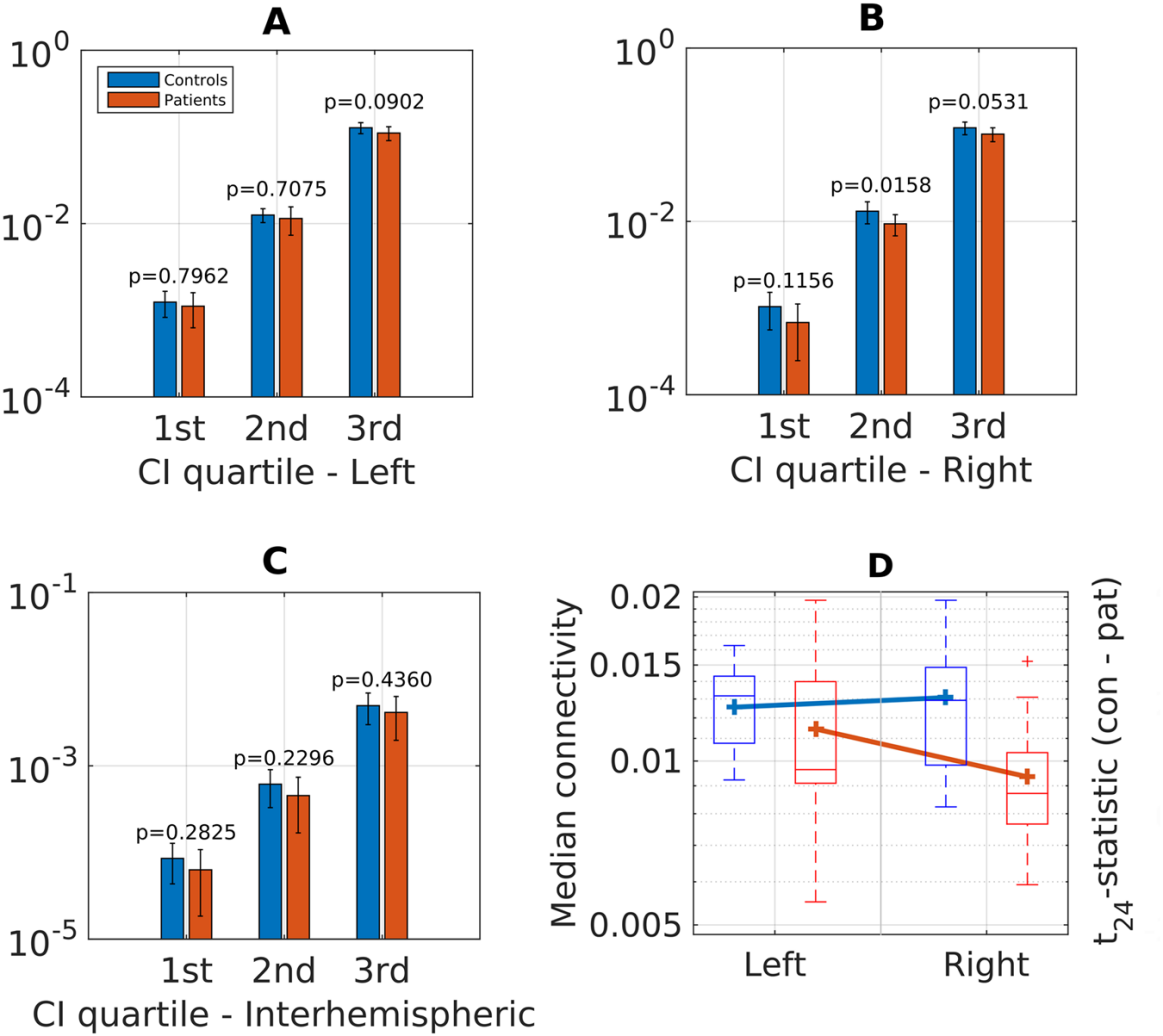
Group differences in overall connectivity. (**A–C**) Group comparison of quartiles (25%, 50% and 75%) of within-subject connectivity strength distributions in individual hemispheres (left, right and interhemispheric). Second (50%) quartile represents median values of connectivity strength. Bar heights indicate group averages, error bars indicate standard deviation. $p$-values are from a ${t}_{max}$-based multiple-comparison resampling test with $n={10}^{6}$ permutations. The group difference in the second quartile (i.e. median) on the right remains significant if corrected across regions ($p=0.0427$). (**D**) Interaction box plot *group* x *hemisphere* for median connectivity strength.

Figure 1A–C displays quartiles of within-subject connectivity strength distributions in the left and right hemispheres, as well as interhemispheric connections. A ${t}_{max}$-based multiple-comparison resampling test with $n={10}^{6}\,$permutations indicated a significantly reduced connectivity in GTS patients relative to healthy controls in the right hemisphere. This finding was corroborated by two-sample Kolmogorov–Smirnov tests of averaged connectivity distributions, which detected significant group differences in the right hemisphere ($p=0.044$) and interhemispheric ($p=0.009$) connections, but not in the left hemisphere ($p=0.820$). Cortical grey matter volumes were similar between left and right hemisphere in patients (left: 254.2 cm³; right 2563 cm³) as well as healthy controls (left 250.9 cm³; right 253.8 cm³). No significant differences in grey matter volumes were detected between both hemispheres in patients and healthy controls (p-values from main effects of group: p = 0.523, hemisphere side: p = 0.591, interaction term group x hemisphere side: p = 0.940).

A 2 × 3 repeated-measures ANOVA of median connectivity strength revealed a large main effect of group (partial${{\rm{\eta }}}^{2}=0.186$), which was statistically significant at the 5% level (${{\rm{F}}}_{1,24}=5.49$, ${\rm{p}}=0.028$). There was also a very large, highly significant main effect of region (${{\rm{F}}}_{1,24}=185.87$, ${\rm{p}} < 0.0001$, ${\eta }_{P}^{2}=0.886$), primarily due to the smaller connectivity strengths in interhemispheric links relative to intrahemispheric connections, reflecting the modular organization of the human brain. The interaction group x region was significant (${F}_{1,24}=3.850$, $p=0.028$) with a large effect size (${\eta }_{P}^{2}=0.138$). Uncorrected post-hoc analyses of simple effects confirmed that the main effect of group was driven by differences in the right hemisphere, where GTS patients showed significantly lower connectivity strength (${{\rm{t}}}_{24}=2.967$,$\,{\rm{p}}=0.007$, Hedges’ ${\rm{g}}=1.127$) compared to healthy volunteers. On the other hand, there was no significant difference between groups in median connectivity strength of the left hemisphere (${{\rm{t}}}_{24}=0.852$, ${\rm{p}}=0.403$, Hedges’${\rm{g}}=0.324$). Analysis of the simple effects of region revealed a disassociation between a non-significant medium-to-large reduction in connectivity on the right in patients (${{\rm{t}}}_{12}=1.844$, ${\rm{p}}=0.090$, Hedges’ ${\rm{g}}=0.593$), and no difference in controls (${{\rm{t}}}_{12}=-0.525$, $p=0.609$, Hedges’ $g=-0.163$), depicted in Fig. 1D.

### Global graph parameters

**Figure 2:**
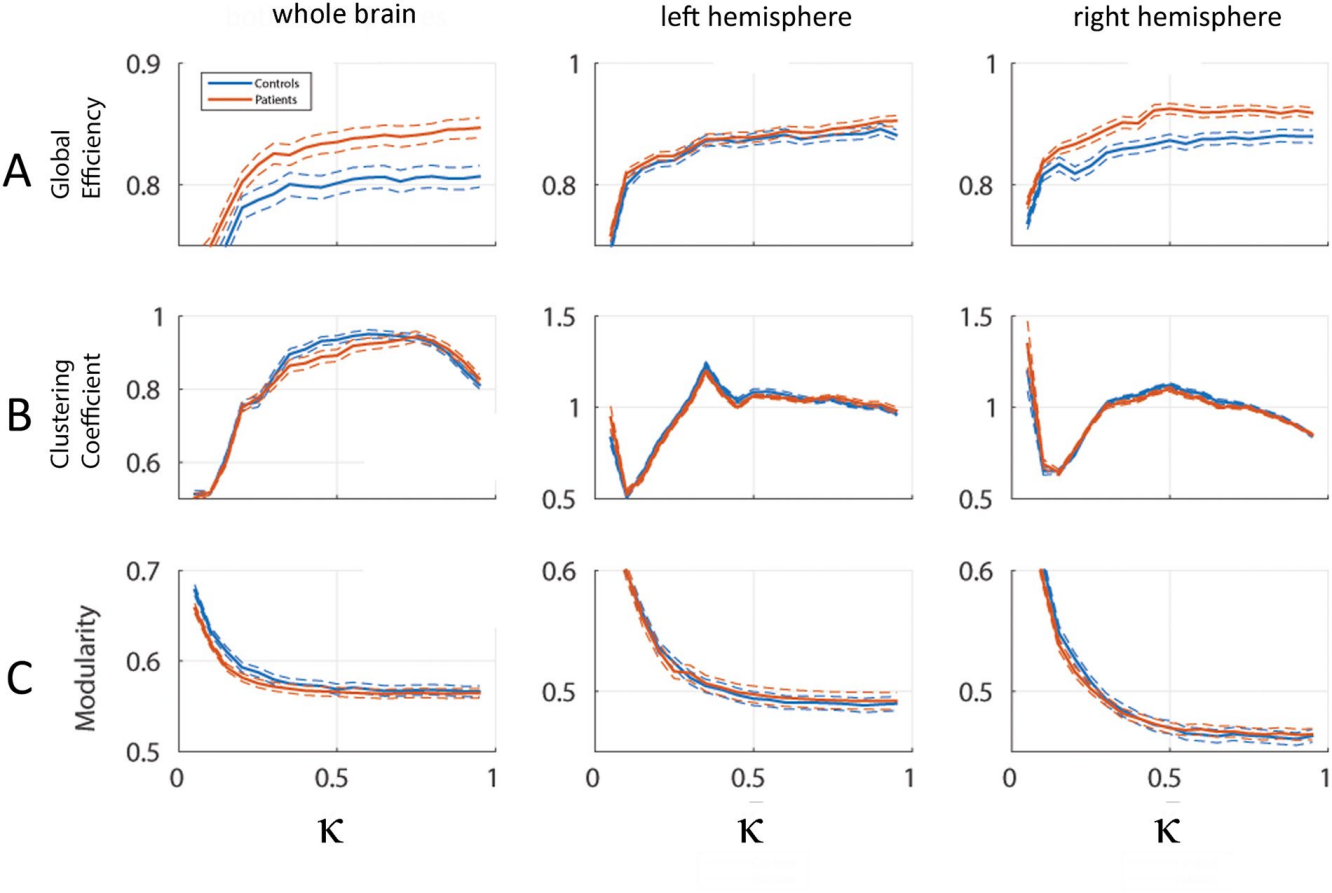
Global graph parameters for healthy controls (blue) and Tourette patients (red) according to hemisphere sides (whole brain, left and right hemispheres). Solid and dashed lines represent group means and standard errors, respectively. Efficiency (**A**) and clustering coefficient (**B**) were normalised with respect to the average of 150 (per subject and parameter) instances of strength- and degree-preserving random reference networks. Clustering has been transformed as $C\mapsto \frac{{C}^{-2}}{\kappa }$for better visualization. Non-normalized values of Modularity (**C**) are shown.

Figure 2 (first column) displays normalised whole-brain network measures for different thresholding parameters$\,\kappa$. The observed higher normalised global efficiency in patients relative to healthy controls was statistically significant. This was shown formally by the MTPC approach with $n={10}^{3}$ permutations which identified the whole $\kappa$-range as a super-critical cluster (${\kappa }_{MTCP}=0.10$, ${t}_{MTCP}=-2.171$, ${A}_{MTCP}=167.07$, ${A}_{crit}=18.60$, $p\le 0.05$). No significant difference was observed for the clustering coefficient and modularity.

Motivated by the left–right differences in overall connectivity strength and the group differences in whole-brain global efficiency, we investigated network characteristics of individual hemispheres (Fig. 2, second and third column). Global efficiency was different between groups for the right-hemispheric network ($n={10}^{3}$, ${\kappa }_{MTCP}=0.15$, ${t}_{MTCP}=-1.84$, ${A}_{MTCP}=126.18$, ${A}_{crit}=11.79$, $p\le 0.05$), but not on the left. For clustering coefficient and modularity, no significant differences were observed.

### Local network measures

Given the finding that group differences in overall connectivity and global graph parameters are limited to the right hemisphere, we further investigated local network characteristics in this subnetwork.

**Figure 3:**
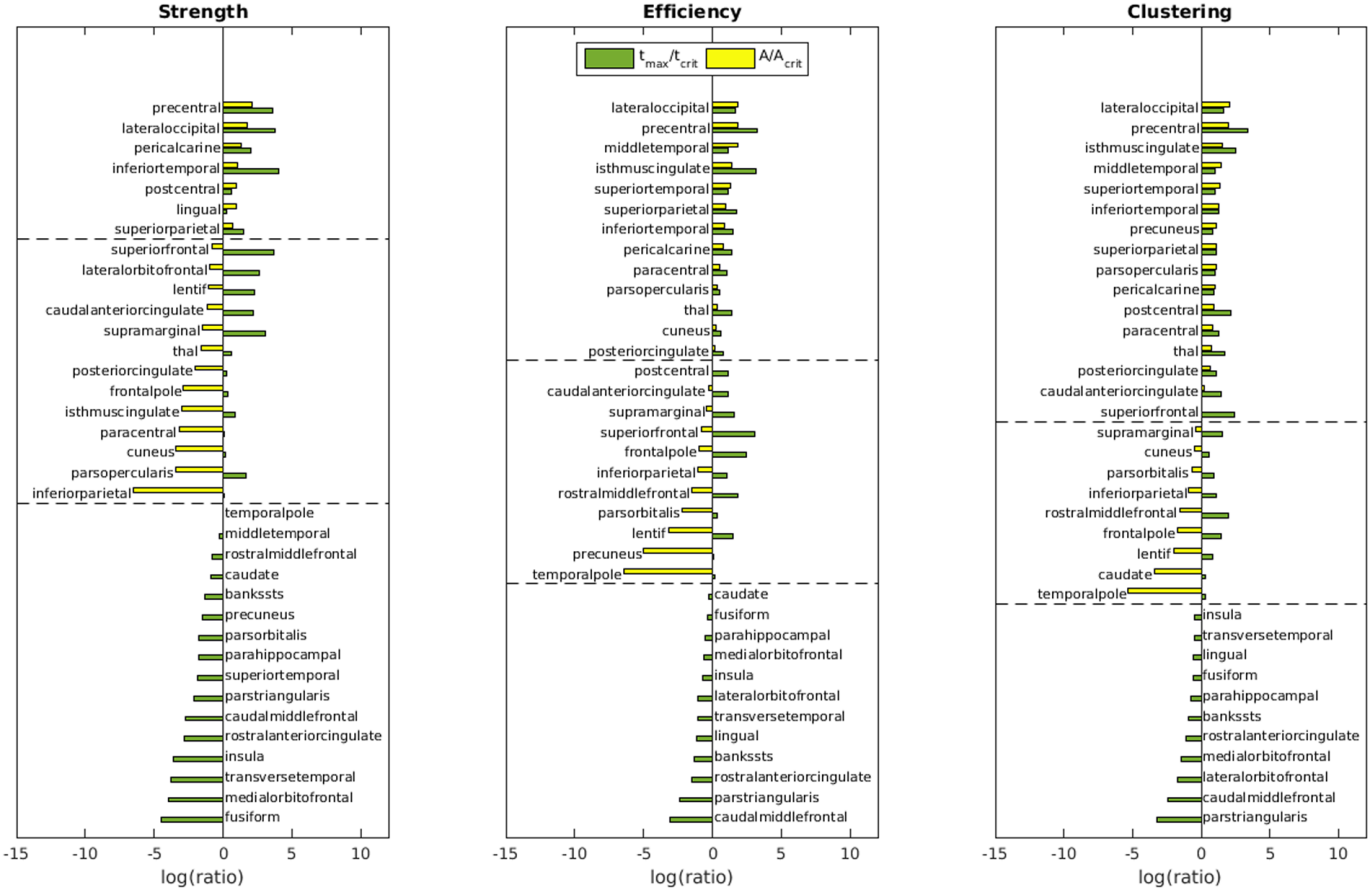
Difference between controls and patients in the local graph parameters strength, efficiency and clustering of different brain regions. Statistical significance is assessed by comparing the maximal $t$-statistic across thresholds, ${t}_{max}$, to the permutations-derived critical $t$-value, ${t}_{crit}$ (green bars). Their ratio exceeding one indicates a group difference with $p < 0.05$. For regions with ${t}_{max} > {t}_{crit}$, effect sizes are further quantified by comparing the supercritical area-under-the curve (scAUC) around the critical threshold to the average scAUC in the permuted data (yellow bars). Note that for regions with ${t}_{max} < {t}_{crit}$, the scAUC is zero.

For thresholding levels$\,\kappa =0.10,\,0.12,\ldots ,\,0.88,\,0.90$, the local graph parameters strength, efficiency and clustering coefficient were calculated for each of 36 brain regions in the right hemisphere. Figure 3 compares, for each brain region, the peak group difference across thresholds, ${t}_{max}$, and the associated super-critical AUC, $A$, with the permutations-derived critical values, ${t}_{crit}$ and ${A}_{crit}$, respectively. The ratio $\frac{{t}_{max}}{{t}_{crit}}$ (green bars) exceeding one indicates that there exists at least one threshold with a significant difference between patients and controls (p < 0.05); if, in addition, the ratio $A/{A}_{crit}$ (yellow bars) is larger than one, this group difference persists over a wider range of thresholds than would be expected by chance. For brain regions satisfying the latter condition, Table 2 reports detailed statistics including values of the local graph parameters at the critical threshold.

**Table 2:** Detailed statistics for brain regions showing a significant group difference in local graph parameters using the MTCP approach.$\,{\kappa }_{MTCP}$ is the threshold with the most significant group difference; columns three and four record the values of the different local graph parameters in controls and patients at this critical value. Hedges’ $g$ and ${t}_{max}$ further quantify the size and significance of the maximal group effect, where positive values indicate a deficit in patients$.\,A\,$is the area-under-the-curve of the supercritical cluster (scAUC) around ${\kappa }_{MTCP}$; it is a numerical measure of the difference between patients and controls that takes into account both the strength of the effect and its persistence across thresholds. Finally, ${t}_{crit}$ and ${A}_{crit}$ are the permutations-derived ($n={10}^{3}$) critical $t$-statistic at 5% significance level and average scAUC, respectively.

Measure	Brain region	${{\boldsymbol{\kappa }}}_{{\boldsymbol{MTCP}}}$	Controls	Patients	Hedges’${\boldsymbol{g}}$	${{\boldsymbol{t}}}_{{\boldsymbol{\max }}}\,$	${{\boldsymbol{t}}}_{{\boldsymbol{crit}}}$	${\boldsymbol{A}}$	${{\boldsymbol{A}}}_{{\boldsymbol{crit}}}$
Strength	precentral	0.28	5.34	4.57	1.15	3.03	0.49	251.88	30.73
	lateraloccipital	0.24	3.38	2.71	0.95	2.51	0.39	176.84	31.11
	inferiortemporal	0.28	5.14	4.16	0.74	1.95	0.26	98.50	33.00
	pericalcarine	0.20	4.56	3.22	0.72	1.91	0.70	80.12	20.67
	superiorparietal	0.34	4.66	4.09	0.61	1.61	0.77	53.73	26.16
	postcentral	0.34	4.76	4.25	0.68	1.78	1.34	30.56	11.65
	lingual	0.34	5.38	4.23	0.61	1.60	1.43	12.05	4.69
Efficiency	precentral	0.30	0.37	0.32	1.12	2.94	0.57	178.25	27.38
	isthmuscingulate	0.54	0.23	0.19	0.92	2.43	0.51	115.60	28.94
	lateraloccipital	0.26	0.32	0.26	0.93	2.45	1.08	114.98	17.63
	superiorparietal	0.34	0.30	0.27	0.74	1.95	0.81	64.78	25.77
	superiortemporal	0.72	0.23	0.20	0.81	2.13	1.20	51.32	14.26
	inferiortemporal	0.80	0.10	0.08	0.68	1.78	0.83	48.64	20.38
	middletemporal	0.80	0.15	0.12	1.01	2.66	1.53	47.73	7.35
	pericalcarine	0.20	0.66	0.49	0.77	2.02	0.99	34.30	15.09
	thalamus	0.34	0.32	0.29	0.61	1.61	0.79	32.59	22.41
	paracentral	0.78	0.13	0.11	0.68	1.80	1.06	28.15	16.81
	posteriorcingulate	0.74	0.19	0.17	0.57	1.49	1.01	21.19	18.63
	cuneus	0.20	0.41	0.33	0.69	1.81	1.32	13.43	10.54
	parsopercularis	0.80	0.15	0.13	0.84	2.20	1.71	7.71	5.27
Clustering	precentral	0.56	0.14	0.12	1.14	3.01	0.54	210.16	27.70
	lateraloccipital	0.52	0.14	0.11	0.96	2.53	1.11	136.51	17.76
	isthmuscingulate	0.48	0.13	0.10	0.94	2.46	0.70	116.79	25.32
	postcentral	0.38	0.32	0.29	0.75	1.97	0.67	59.91	24.75
	inferiortemporal	0.80	0.06	0.05	0.75	1.97	1.05	59.20	16.01
	superiorparietal	0.58	0.09	0.08	0.74	1.96	1.15	57.06	18.65
	thalamus	0.80	0.11	0.10	0.62	1.63	0.70	50.37	23.97
	superiortemporal	0.80	0.12	0.11	0.83	2.18	1.30	49.76	12.53
	paracentral	0.78	0.09	0.07	0.70	1.85	0.99	40.22	17.97
	middletemporal	0.80	0.08	0.07	0.89	2.35	1.38	39.87	9.40
	precuneus	0.62	0.10	0.09	0.76	2.00	1.32	37.89	12.25
	posteriorcingulate	0.48	0.24	0.21	0.65	1.71	1.00	37.48	18.61
	superiorfrontal	0.80	0.09	0.08	0.57	1.49	0.44	33.14	30.81
	caudalanteriorcingulate	0.48	0.24	0.21	0.53	1.39	0.68	32.85	25.67
	pericalcarine	0.20	0.64	0.48	0.72	1.90	1.18	30.18	10.48
	parsopercularis	0.80	0.09	0.08	0.88	2.33	1.42	23.65	8.14

As shown in Table 2, significant reductions of strength, efficiency and clustering coefficient were observed in patients for somatosensory (postcentral), motor (precentral), frontal (pars opercularis), occipital (laterooccipital) and cingulate (isthmus, posterior) areas. In addition, less pronounced reductions of these local graph parameters were found in temporal (inferior, middle, superior) and occipital (lingual, pericalcarine) regions as well as the thalamus. None of the examined local graph parameters was significantly increased in GTS patients.

### Correlation with clinical score

**Figure 4:**
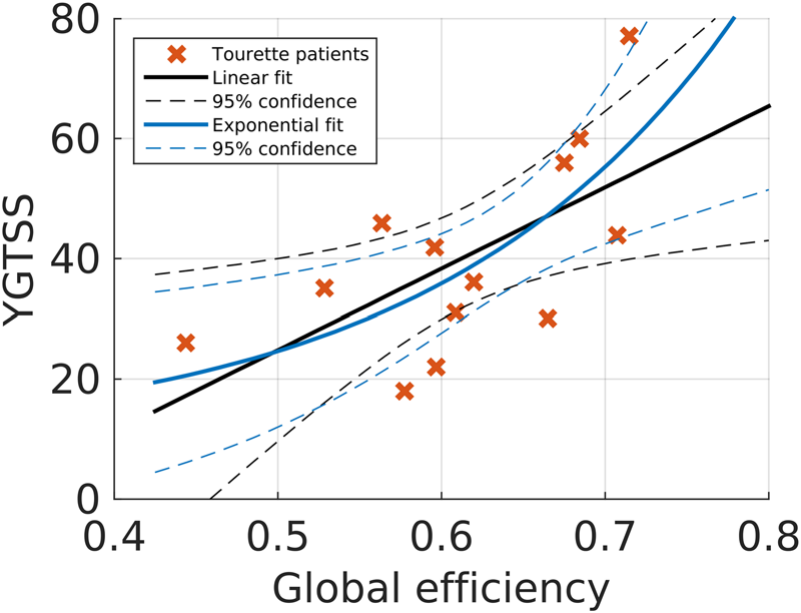
Correlation plot of the relation between YGTSS and non-normalised global efficiency in the right hemisphere (R = 0.6262, p = 0.022). Lines of best fit (solid) and 95% non-simultaneous functional confidence bands (dashed) for linear and two-parameter exponential models. Crosses indicate measurements of individual patients.

A significant positive linear correlation between the non-normalised global efficiency of the non-thresholded right-hemispheric network and YGTSS was found ($R=0.63$, $p=0.022$; see Fig. 4). The effect persisted when age was included as a covariate (partial linear correlation coefficient$\,{R}_{\partial }=0.61$, $p=0.037$). Statistics based on rank correlations were similar ($R=0.60$, $p=0.032$;$\,{R}_{\partial }=0.63$, $p=0.027$). The convexity of the scatter plot suggests a nonlinear relation between efficiency and YGTSS. Indeed, the relative likelihood of a two-parameter exponential model (root-mean-square-error (RMSE) 12.5, adjusted R^2^ 0.44, F-statistic vs. constant model 10.3, p-value 0.008, Akaike Information Criterion (AIC_c_) 103.49) with respect to a simple linear model (${\rm{RMSE}}=13.5$, ${{\rm{R}}}_{{\rm{adj}}}^{2}=0.34$, ${{\rm{F}}}_{1,11}=7.09$, ${\rm{p}}=0.022$, ${{\rm{AIC}}}_{{\rm{c}}}=105.62$) is equal to 2.91.

No significant correlation was observed between YGTSS and non-normalised global efficiency of the left-hemispheric network (Linear – $R=0.50$, $p=0.084$; ${R}_{\partial }=0.38$, $p=0.225$. Rank – $R=0.54$, $p=0.061$; ${R}_{\partial }=0.46$, $p=0.137$). There was no significant correlation between YGTSS and any of the other global network measures (normalised efficiency, non-/normalised clustering, modularity) considered earlier. There was also no significant correlation between YGTSS and median connectivity strength or any of the local network measures.

## Discussion

Our study of structural brain networks in a group of adult patients with Gilles de la Tourette syndrome yielded three major findings. Firstly, we found a significant reduction in overall connectivity strength in the right hemisphere of GTS patients affecting the majority of connections. This was accompanied by an overall reduction in local graph measures such as local clustering and local efficiency reflecting decreased segregation of structural subnetworks. Second, contrasting these findings, we observed a higher global integration of right-hemispheric structural networks by graph theoretical analysis in terms of higher normalized global efficiency in GTS patients as compared to the age-matched control group. Finally, increased global efficiency was correlated with disease severity as measured by the YGTSS indicating the clinical relevance of network topology alterations observed in our analysis.

Tics are the defining clinical feature and hallmark of GTS. Up to date, understanding the exact structural and functional basis of tics still remains elusive, although involvement and dysfunction of widely distributed, cortical and subcortical networks have been proposed^12, 40^. From a structural point of view, alterations of both grey and white matter have emerged as a common theme in previous neuroimaging studies including children and adults with GTS. These changes include reduction in white matter volumes in primary and secondary motor areas^41^, microstructural changes of widely distributed, fronto-subcortical circuits^42^ or reduced cortical grey^43^ and white matter volumes and structural integrity of the underlying white matter extending beyond the primary motor pathway^44^ associated with clinical severity. Furthermore, irregularly reduced white matter integrity underlying the somatosensory cortices have been previously detected in an adult group of patient with GTS by diffusion tensor imaging^45^. In accordance with these observations, our results indicate disruption of structural networks and decreased strength in connectivity most likely resulting from microstructural changes of subcortical white matter tracts.

We have previously examined this group of patients by applying DTI and reported reduced connectivity in two thirds of white matter tracts in a predefined and specific network of motor control^24^. In the current study, we extend our analysis beyond a highly localized and functionally defined subnetwork to a much larger, global brain network based on an established cortical parcellation scheme^29, 30^. To the best of our knowledge, this is the first study to report results from graph theoretical analysis of structural brain networks in patients with GTS. By this approach, we identified reduced values of local graph measures indicating abnormally altered structural network topology exceeding previously reported connectivity deficits: local efficiency is a measure of the average structural integrity within adjacent subgraphs and thus illustrates the efficiency of communication among the immediate neighbours of a node if it is removed from the network. Decreased local efficiency of a node thus reveals impairment of connectivity within its immediate local community. Together with reduced local clustering, reduction of local graph measures in patients with GTS point to an altered network topology that is less robust and resilient against random network changes^14, 31^. Our findings indicate the presence of a less segregated structural network and point to a reduced potential for functional segregation and information transfer. These findings were most prominent in local subnetworks associated with the precentral gyrus (Table 2, Fig. 3), which corresponds well with our previous findings of reduced node-to-node connectivity involving motor and premotor areas^24^. In line with these findings, a recent study of 27 children with GTS showed widespread alterations of white matter integrity underlying the pre- and postcentral cortex associated with clinical severity^44^. Similarly, reduced values of FA were detected involving subcortical motor pathways in 19 unmedicated adult patients with GTS^42^. Wen *et al*. also reported reductions of FA at the cingulate^44^, a region shown to be activated during tic inhibition and demonstrating strong structural interconnections with prefrontal, orbitofrontal, subcortical and motor areas^46^. Interestingly, reduced local efficiency and clustering were also shown at the cingulate in our study. To a lesser extent, disturbed local networks have been found in our study involving the pars opercularis of the inferior frontal gyrus. Previous neuroimaging studies have demonstrated this area (Brodman area 44) to be related to hand movements^47^ and highlighted its role as a supplemental motor area during preparation and execution of manual tasks. Lastly, we observed reduced local efficiency in subnetworks involving temporal and temporo-occipital areas. Although the role of the temporal lobe in tic generation remains less certain, results of previous studies using structural^48, 49^ and functional^46^ MRI indicate that various temporal regions are involved in the control of tics. Taken together, these findings conjointly with our results indicate local network disturbances and potentially impaired specialization of information transfer in a group of cortical areas involved in planning, controlling and execution of motor functions.

In addition to disturbed local networks, we observed significantly higher normalized global efficiency in right-hemispheric structural brain networks of patients with GTS. Whereas local graph parameters primarily indicate changes in short range subnetworks, the graph theoretical measure of global efficiency is based on minimum path lengths, primarily between distant nodes or brain areas connected by long association tracts. In contrast to focal changes in structural connectivity affecting local graph measures, global efficiency is more likely affected by a relative diffuse alteration of white matter. In addition, normalization of global efficiency with respect to reference networks with equal connectivity profiles removes confounding effects of individual edge strengths between groups. Thus, it relates closely to overall network topology, where higher values indicate increased structural integration of distributed brain regions. Comparability of our findings with previous results is limited due to the absence of similar studies analysing graph theoretical properties of structural brain networks in GTS patients. However, there are few observations pointing to cerebral plasticity beyond primary neurodegeneration: Abnormally enhanced connectivity in cortico-subcortical networks has been reported in a recent study of 49 adult GTS patients with obsessive compulsive disorder using diffusion tensor imaging. Interestingly, the authors have previously reported increased functional integration of the blood oxygen level-dependent signal in the identical group of patients examined by resting state fMRI^19^, which would suggest stronger functional interactions among separate brain regions. This finding appears complimentary to our finding of increased global efficiency or overall reduced path lengths which might facilitate functional integration and faster information transfer between specialized distributed brain regions^31, 50^.

Graph theoretical analysis revealed altered structural networks in the right hemisphere of our patients. All patients were right-handed and there was no obvious lateralization in their tics by clinical observation. There also were no significant differences in volumes of grey matter between left and right hemispheres that technically could act as a confounding factor explaining this lateralized finding. While no general concept of lateralized pathology has emerged from previous structural neuroimaging studies of GTS patients, there are numerous examples of asymmetrically distributed changes of basal ganglia^51, 52^, and cortical volumes^42, 53^, as well as white matter integrity^48^. Neuroimaging studies have also yielded lateralized results with increased functional connectivity of the right insula as well as altered right-hemispheric properties of a cortico-subcortical “urge and tic network” in resting state MRI^54^ and activations in a right-lateralized network representing efforts of inhibitory control during response inhibition^55, 56^. Results of deep brain stimulation in patients with GTS support the notion that bilateral effects in terms of tic reduction can be achieved by unilateral stimulation^57^. Taken together, these findings suggest the presence of lateralized brain functions involved in tic generation and control in GTS, which would be in accordance with hemispheric differences in topological organization of structural brain networks.

Findings from structural and functional neuroimaging have previously supported the concept of GTS as a disorder of neuronal development and maturation. Resting-state fMRI has shown functional interactions between cortico-subcortical regions in adult GTS patients resembling networks of young children^58^. Disorganized and “functional immature” sensorimotor and pre-motor networks were furthermore revealed in adult patients by Worbe *et al*.^19^. On a microscopic level, these finding have been underpinned by post-mortem neuropathological studies demonstrating widespread disturbances in neuronal maturation^59^. The observation of a widespread reduction in connection strength with reduced segregation of structural networks involved in execution and control of motor function in our sample might also result from impaired neuronal integrity due to delayed or incomplete neuronal maturation since connectivity as measured by DTI-tractography relates to structural properties of white matter tracts such as the degree of myelination, number of axons, degree of axonal pruning and the cohesiveness or truncation of axons^60^. Findings supporting this hypothesis have recently been reported by applying graph theoretical approaches demonstrating delayed stabilization and individualization of structural brain connectomes in individuals with psychiatric disorders^61^.

During normal structural brain development from childhood to adulthood, white matter networks in the right and left hemisphere appear to follow different trajectories of structural maturation and connectivity. In the largest structural study of network topology to date, Dennis and colleagues observed diverging linear changes of global graph parameters in 439 subjects between the age of 12 to 30 in a cross-sectional design^62^. Whereas increasing global efficiency were observed in the left hemisphere, the right hemisphere demonstrated an opposing trend of linear decreases in global efficiency. In contrast to this trajectory, a significantly increased global efficiency was observed at the right hemisphere in our patients, a finding that might be understood as disturbed or immature development of structural brain network organization and thus a primary marker of the pathology of GTS. On the other hand, increased right hemispheric global efficiency might reflect a compensatory mechanism representing the brains response to altered structural connectivity and impaired local network organization by adaptions of higher order topologic networks characteristics. The observation of a correlation of increased global efficiency with disease severity points towards the clinical relevance of network organizations changes but does not help in answering the question whether the observed changes reflect a primary pathology or adaptive plasticity during the course of the disease. In light of the developmental trajectories of network measures reported by Dennis *et al*., these findings could be viewed as a primary disease marker of abnormally increased structural integration in our patients leading to higher frequency of tics. On the other hand, training effects of continued tic inhibition have been proposed previously leading to enhanced inhibitory abilities in patients with GTS that might be more pronounced in patients with more severe disease^63^. As we do not have longitudinal data these observations to remain to be validated in future work.

Beside the lack of longitudinal data, there are additional limitations of our study that have to be addressed. First, the small sample size restricts the generalizability of our results. Second, diffusion tensor imaging and probabilistic tractography allow reconstruction of weighted connectome edges, that are not a direct quantification of neuronal fibre counts but rather a computational model representation and abstraction of the actual trajectories subjected to limitations of data acquisition and processing as described previously^64^.

In conclusion, our network analysis of structural connectomes in a group of adult GTS patients demonstrated disturbances of structural integrity of networks of right hemispheric cortical areas involved in planning, execution and control of motor functions. This is contrasted by the observation of increased global efficiency and structural integration at the network level which correlated with clinical severity of tics. This finding might reflect a primary pathology with an imbalance of segregation and integration of structural brain networks. Increased global efficiency might also indicate adaptive plasticity of the brain responding to structural brain network changes in Gilles de la Tourette syndrome.
